# Acute Kidney Injury in Critically Ill Children Is Not all Acute: Lessons Over the Last 5 Years

**DOI:** 10.3389/fped.2021.648587

**Published:** 2021-03-15

**Authors:** Erin Hessey, Nabil Melhem, Rashid Alobaidi, Emma Ulrich, Catherine Morgan, Sean M. Bagshaw, Manish D. Sinha

**Affiliations:** ^1^Faculty of Medicine & Dentistry, University of Alberta, Edmonton, AB, Canada; ^2^Department of Pediatric Nephrology, Evelina London Children's Hospital, London, United Kingdom; ^3^Division of Pediatric Critical Care, Department of Pediatrics, University of Alberta and Stollery Children's Hospital, Edmonton, AB, Canada; ^4^Department of Pediatric Nephrology, Stollery Children's Hospital, Edmonton, AB, Canada; ^5^Department of Critical Care Medicine, Faculty of Medicine and Dentistry and Alberta Health Services—Edmonton Zone, University of Alberta, Edmonton, AB, Canada; ^6^Alberta Critical Care Strategic Clinical Network, Alberta Health Services, Edmonton, AB, Canada; ^7^King's College London, London, United Kingdom

**Keywords:** acute kideny injury, chronic kidney disease, long-term follow up, hypertension, healthcare utilization, mortality, critical care

## Abstract

Acute kidney injury (AKI) in the pediatric intensive care unit (PICU) is an important risk factor for increased morbidity and mortality during hospitalization. Over the past decade, accumulated data on children and young people indicates that acute episodes of kidney dysfunction can have lasting consequences on multiple organ systems and health outcomes. To date, there are no guidelines for follow-up of surviving children that may be at risk of long-term sequelae following AKI in the PICU. This narrative review aims to describe literature from the last 5 years on the risk of medium and long-term kidney and non-kidney outcomes after AKI in the PICU. More specifically, we will focus on outcomes in children and young people following AKI in the general PICU population and children undergoing cardiac surgery. These outcomes include mortality, hypertension, proteinuria, chronic kidney disease, and healthcare utilization. We also aim to highlight current gaps in knowledge in medium and long-term outcomes in this pediatric population. We suggest a framework for future research to develop evidence-based guidelines for follow-up of children surviving an episode of critical illness and AKI.

## Introduction

Acute kidney injury (AKI) is defined as an abrupt onset of kidney dysfunction. AKI occurs in 20–30% of the general pediatric intensive care (PICU) population and up to 50% in children following cardiac surgery (1–6). Compared to children who do not develop AKI in the PICU, children with AKI are at higher risk of poor early outcomes, including mortality, receipt and longer duration of mechanical ventilation, and prolonged length of PICU and subsequent hospital stay (7–10).

More recently, research has focused on evaluating the medium and long-term sequelae of AKI sustained in the PICU. One of the largest early follow-up studies found that 10% of children with AKI in the PICU developed chronic kidney disease (CKD) [defined as glomerular filtration rate (GFR) <60 ml/min/1.73 m^2^ or albuminuria] 1–3 years later (11). Additionally, 47% of children with AKI were considered to be at risk of CKD [defined as GFR 60–90 ml/min/1.73 m^2^, hypertension, and/or hyperfiltration (GFR ≥ 150 ml/min/1.73m^2^)]. In 2014, Greenberg et al. performed a systematic review and meta-analysis to examine the long-term kidney outcomes after AKI (12). These early studies found that children with AKI had higher rates of hypertension, proteinuria, CKD, and mortality relative to the general pediatric population. However, the authors highlighted that there was significant heterogeneity between studies with an absence of contemporaneous patients with no AKI, and variable definitions of AKI, which together made it difficult to evaluate the association between AKI and outcomes. These issues have been addressed systematically in recent studies standardizing AKI definitions, with large study cohorts that include non-AKI comparison groups, and an overall increase in the quality of research and rigor of analyses. This has allowed for a better understanding of the association between AKI and long-term outcomes.

The objectives of this narrative review are to: (i) summarize literature over the past 5 years for relevant medium and long-term outcomes following AKI in the general PICU population and children undergoing cardiac surgery; (ii) highlight risk associations for reported adverse outcomes; and (iii) discuss future directions for clinical guidelines and research to mitigate adverse outcomes in this high-risk population.

## Mortality

Previous studies evaluating short- and long-term mortality following AKI lacked non-AKI comparison groups, however, they uniformly highlight that mortality rates in patients with AKI were higher than in the general population (12, 13). Since 2015, we identified eight studies that examined the association of AKI with mortality and compared this to children who did not develop AKI, with follow-up ranging from 28-days to 5–7 years (Table 1) (3, 6, 14–19). As highlighted in Table 1, despite differences in study populations and duration of follow-up, all studies reported higher mortality in those with AKI when compared with children who did not experience AKI whilst in the PICU, independent of illness severity and other important confounders.

**Table 1:** Summary of studies evaluating the association between AKI and mortality published over the past 5-years.

**References**	**Study design**	***N* patients**	**Inclusion criteria**	**AKI definition**	**Duration of follow up**	**Outcome definition**	**Findings**
Sanchez-Pinto et al. (14)	Single center retrospective cohort study	8,260	General PICU, non-cardiac surgery	KDIGO	28-days	Chart review, includes in-hospital mortality	• In adjusted analysis both resolved and persistent AKI was associated with 28-day mortality
Al-Otaibi et al. (15)	Single center retrospective cohort study	131	General PICU	pRIFLE	2-years	Chart review, includes in-hospital mortality	• 40% 2-year mortality in patients with AKI • In adjusted analysis stage 3 (failure) was not statistically significantly associated with 2-year mortality
Hirano et al. (16)	Single center retrospective cohort study	418	Cardiac surgery	pRIFLE	2-years	Prospective patient database, includes in-hospital mortality	• 23 of 104 (22%) patients with AKI died during 2-year follow-up • AKI was associated with 2-year mortality [aHR 7.47 (2.88–19.40)]
Kaddourah et al. (3)	Multinational, prospective observational cohort study	4,984	General PICU	KDIGO	28-days	Chart review, includes in-hospital mortality	• 60 of 543 patients (11%) with severe AKI died compared to 105 of 4,140 patients (2.5%) without severe AKI (*P* < 0.001) • Severe AKI associated with increased risk of death [aOR 1.77 (1.17–2.68)]
Hessey et al. (17)	Two center retrospective cohort study	2,041	General PICU, non-cardiac surgery	KDIGO	5–7 years	Administrative data, excludes in-hospital mortality	• AKI was associated with over 3 × higher risk of 5–7 year mortality • AKI was not associated with 30-day or 1-year mortality when hospital mortality excluded
Alobaidi et al. (6)	Multicenter retrospective cohort study	1,017	General PICU	KDIGO	1-year	Administrative data, includes in-hospital mortality	• 56 (5.5%) of patients died within 1 year of PICU admission • Severe AKI associated with greater 1-year mortality [aOR 5.50 (2.76–10.96)] • Post-discharge mortality (*n* = 18) was higher in patients with severe AKI [aOR 2.84 (1.04–7.81)]
Nunes et al. (18)	Multicenter prospective cohort study	402	Cardiac surgery	KDIGO	1-year	Chart review, includes in-hospital mortality	• Severe AKI associated with increased risk of 30-day mortality [aHR 11.7 (1.9–72.6)] • At 1-year 4 of 344 (1.2%) non-AKI/stage 1 and 5 of 58 (8.6%) stage 2 or 3 AKI died
Zhang et al. (19)	Single center retrospective cohort study	80	Liver transplantation	KDIGO	3-year	Chart review, includes in-hospital mortality	• The 3-year survival was higher in non-AKI patients (95%) compared to AKI patients (87%) but did not reach statistical significance

*Key articles evaluating the association between PICU-AKI and long-term mortality published since 2015. This may not be an exhaustive list as a formal systematic review search was not performed*.

*KDIGO, Kidney Disease: Improving Global Outcomes; pRIFLE, pediatric Risk, Injury, Failure, Loss, End stage renal disease; AKI, acute kidney injury; aOR, adjusted odds ratio; aHR, adjusted hazard ratio; PICU, pediatric intensive care unit; CKD, chronic kidney disease; SCr, serum creatinine*.

### General PICU Population

In a multinational study of 4,984 general PICU patients, Kaddourah *et al*. showed that severe AKI [defined throughout this review as stage 2 or 3 AKI by Kidney Disease: Improving Global Outcomes (KDIGO) criteria unless specified] was associated with 77% greater odds of 28-day mortality following adjustments for risk factors that differed between survivors and non-survivors including admission diagnoses, comorbidities, illness severity, and PICU interventions (3). Similarly, when compared to those with no AKI, a large multicenter Canadian study observed that severe AKI was associated with over 5 times greater risk of mortality 1-year after PICU admission following adjustment for illness severity and other important confounders (6). It is important to highlight that both these studies included in-hospital mortality from initial admission in their outcomes. In the Canadian study 52 (5.5%) patients died, 18 of whom died after discharge. Severe AKI was still associated with post-discharge mortality [odds ratio [OR] (95% confidence interval [CI]): 2.84 (10.4–7.81)] (6).

The longest follow-up study looked at mortality 5–7 years after hospital discharge in a non-cardiac surgery PICU population and found that AKI was associated with over 3 times higher risk of mortality (17). Interestingly, this paper demonstrated that the association between AKI and 30-day and 1-year mortality was conditional on the inclusion of hospital mortality in the outcome. When hospital mortality was included, AKI was associated with 30-day and 1-year mortality with a similar magnitude of association as reported in previous studies (3, 6, 17).

### Cardiac Surgery Population

Two studies specifically focused on mortality in children with AKI following cardiac surgery. In a multicenter study of over 400 patients, Nune et al. found that children with severe AKI post-cardiac surgery had over 11 times higher risk of mortality 30-day after surgery, following adjustment for age, surgical risk score, cardiopulmonary bypass time, and cyanotic heart disease (18). In a similar-sized population, Hirano et al. reported a higher risk of 2-year mortality in children with AKI [defined by Pediatric Risk, Injury, Failure, Loss, End Stage Renal Disease (pRIFLE) criteria] post-cardiac surgery [adjusted hazard ratio (95% CI): 7.47 (2.88–19.40)] (16). Both of these studies included hospital mortality from the initial cardiac surgery admission.

### What's New?

Since the publication of the systematic review by Greenberg et al. we now have comparable groups (with and without AKI during hospitalization) that allow us to evaluate the association between AKI and mortality (12). All studies found that children who develop AKI in the PICU had higher mortality, independent of illness severity measures, compared to patients admitted to the PICU who did not develop AKI. However, it is important to highlight that many of these studies included in-hospital mortality from the index admission.

## Hypertension, Proteinuria, and Chronic Kidney Disease

In adult studies, AKI in the ICU is independently associated with significantly worse long-term kidney outcomes including hypertension, proteinuria, and CKD (20–22). The potential mechanism for the progression of AKI to CKD includes loss of kidney mass during the acute event resulting in progressive hyperfiltration damage, glomerulosclerosis, and fibrosis (23). This relationship has been challenging to study in the pediatric population as a result of the known physiologic maturation in glomerular filtration rate following birth, the current lack of consensus guidelines on how best to monitor these children over time resulting in varying follow-up practices, and inconsistent definitions of long-term kidney outcomes in the literature. Initial findings showed a higher prevalence of adverse kidney outcomes following AKI compared to the general population; however, in these studies subject numbers were small, follow-up duration was limited, and many lacked non-AKI comparison groups (12). Since 2015 we found eleven studies examining the association between PICU-AKI and hypertension, proteinuria, and CKD (Table 2) (2, 4, 15, 24–32).

**Table 2:** Summary of studies evaluating the association between AKI and long-term kidney outcomes published over the past 5-years.

**References**	**Study design**	***N* patients**	**Inclusion criteria**	**AKI definition**	**Duration of follow up**	**Outcome definition**	**Findings**
Cooper et al. (24)	Single center retrospective cohort study	51	Cardiopulmonary bypass surgery	pRIFLE	7 years	(i) low eGFR (<90 ml/min/1.73 m^2^) (ii) albuminuria (ACR >30 mg/g) (iii) Hypertension (≥90th percentile)	• No significant association between outcome measures of CKD and PICU-AKI after CPB surgery
Hollander et al. (2)	Single center retrospective cohort study	88	Heart transplant recipients	KDIGO	1 year	(i) eGFR <60 mL/min/1.73 m^2^ for more than 3 months	• 5% of population developed CKD • Non-recovery from AKI associated with CKD
Al-Otaibi et al. (15)	Single center retrospective cohort study	131	General PICU	pRIFLE	2 years	(i) Hypertension (>95th percentile) (ii) Proteinuria (PCR > 30 mg/dl) (iii) Reduction in GFR	• PICU patients with AKI had high prevalence of CKD (33%) and hypertension (73%)
Madsen et al. (4)	Multicenter retrospective cohort study	382	Cardiac surgery	KDIGO	5 years	(i) low eGFR (<90 ml/min/1.73 m^2^)	• AKI associated with an increased risk for CKD [aHR 3.8 (1.4–10.4)]
Greenberg et al. (25)	Single center prospective cohort study	110	Cardiopulmonary bypass surgery	AKIN	5 years	(i) low eGFR (<90 ml/min/1.73 m^2^) (ii) albuminuria (ACR > 30 mg/g) (iii) Hypertension (≥95th percentile)	• No significant association between outcome measures of CKD and AKI
Hessey et al. (26)	Two center retrospective cohort study	2,235	General PICU	KDIGO	5 years	(i) ≥1 CKD diagnostic code or ≥1 CKD-specific medication prescription	• Patients with AKI had increased risk of a CKD diagnosis
Benisty et al. (27)	Two center prospective cohort study	277	General PICU, non-cardiac surgery	KDIGO	6 years	(i) low eGFR (<90 ml/min/1.73 m^2^) (ii) albuminuria (ACR>30 mg/g) (iii) BP ≥90th percentile	• AKI and stage 2 or 3 AKI associated with 2.2- and 6.6-fold higher adjusted odds of CKD and pre-hypertension or worse
Hessey et al. (28)	Two center retrospective cohort study	1,978	General PICU, non-cardiac surgery	KDIGO	5 years	(i) ≥1 hypertension diagnostic code or ≥1 hypertension-specific medication prescription	• Patients with AKI and stage 2 or 3 AKI had increased risk of a hypertension diagnosis
Huynh et al. (29)	Two center retrospective cohort study	58	Neonatal cardiac surgery	KDIGO	6 years	(i) low eGFR (<90 ml/min/1.73 m^2^) (ii) albuminuria (ACR>3 mg/mmol) (iii) BP ≥90th percentile	• Cardiac surgery associated AKI was not associated with CKD or hypertension
Menon et al. (30)	Single center retrospective cohort study	221	Heart transplant recipients (*n* = 109) Liver transplant recipients (*n* = 112)	KDIGO	5 year	(i) low eGFR (<60 ml/min/1.73 m^2^)	• No difference in incidence of CKD amongst heart transplant recipients with PICU-AKI vs. no AKI • Liver transplant recipients with PICU-AKI showed a higher risk of CKD compared to those with no AKI
Zappitelli et al. (31)	Two center prospective cohort study	124	Cardiac surgery	KDIGO	4 years	(i) low eGFR for age (ii) albuminuria (ACR > 30 mg/g) (iii) Hypertension (≥95th percentile)	• AKI not associated with CKD and hypertension at follow-up

*Key articles evaluating the association between PICU-AKI and long-term kidney outcomes including CKD, proteinuria, and hypertension published since 2015. This may not be an exhaustive list as a formal systematic review search was not performed*.

*KDIGO, Kidney Disease: Improving Global Outcomes; pRIFLE, pediatric Risk, Injury, Failure, Loss, End stage renal disease.; AKIN, acute kidney injury network criteria; AKI, acute kidney injury; aHR, adjusted hazard ratio; PICU, pediatric intensive care unit; CKD, chronic kidney disease; SCr, serum creatinine; eGFR, estimated glomerular; CPB, cardiopulmonary bypass*.

### General PICU Population

There have been four studies examining the association of CKD following AKI in general PICU cohorts. The follow-up time across these studies varied from 2 to 6 years, as did the methods of defining outcomes. However, consistently across all studies patients with mixed etiology, PICU-AKI were at higher risk of developing hypertension, proteinuria, and/or CKD over the long-term (15, 26–28). Two studies in cohorts of general PICU-AKI patients measured outcomes using laboratory defined CKD (albumin/creatinine >30 mg/g or GFR <90 ml/min/1.73 m^2^) or measured office blood pressure abnormalities (15, 27). The prevalence of CKD and hypertension was high in the AKI population in both studies. One of these studies by Benisty et al. found that 6 years after PICU admission any AKI (i.e., stage 1, 2, or 3 by KDIGO criteria) and severe AKI (stage 2 or 3) were associated with a 2.2 [95% CI: 1.1–4.4] and 6.6 [95% CI: 1.5–28.3] higher adjusted odds ratio for CKD and pre-hypertension or worse, respectively (27).

Two studies from the same cohort in Quebec alternatively used administrative data (diagnostic and medication codes) to define CKD and hypertension outcomes (26, 28). In this large two-center retrospective cohort of 2,245 subjects, even following mild (stage 1) AKI, children had a significantly increased risk of CKD diagnosis after a 5-year follow-up period (26). At follow-up, 2% had a diagnosis of CKD. Those with mild AKI (stage 1) had an increased risk of CKD with an adjusted hazard ratio of 2.2 [95% CI:1.1–4.5]. Those with more severe AKI (stage 2 or 3) had a higher risk of CKD with an adjusted hazard ratio of 2.5 [95% CI: 1.1–5.7] (26). Looking exclusively at hypertension diagnosis based on administrative data in the same large two-center cohort, Hessey et al. found that patients with PICU-AKI had over twice the risk of a hypertension diagnosis with an adjusted hazard ratio of 2.19 [95% CI: 1.47–3.26] (28).

### Cardiac Surgery Population

Six studies reported long-term kidney outcomes following PICU-AKI exclusively in subjects who underwent cardiac surgery (2, 4, 24, 25, 29, 31). Unlike the general PICU population, there is conflicting evidence on whether AKI after cardiac surgery is associated with negative long-term kidney outcomes. A two-center prospective cohort study of 124 children undergoing cardiac surgery found no association between AKI and CKD or hypertension after 2 years (31). The overall prevalence of CKD and hypertension was high at 20 and 30%, respectively, and young age at surgery was the only factor associated with CKD development. Contradicting these findings, in a single-center retrospective cohort study of 382 subjects who had undergone congenital cardiac surgery, after a median 3-year follow-up period, 11% of patients with AKI demonstrated CKD (4). The hazard ratio for CKD development amongst those with AKI compared with the non-AKI group was notably high, at 3.8 [95% CI: 1.4–10.4] following adjustment for sex, age, and surgical complexity (4). Similarly, two studies looking at long-term kidney outcomes exclusively in subjects who had undergone cardiopulmonary bypass surgery did not find an association between AKI and CKD (GFR <90 ml/min/1.73 m^2^ or albuminuria) or hypertension (blood pressure >95th percentile) (24, 25).

In a cardiac sub-group of 88 heart transplant recipients, advanced CKD (GFR <60 mL/min/1.73 m^2^) developed in 5% of the population after 1-year follow-up and was more common in those who did not fully recover kidney function and in those with more severe AKI (stage 2 or 3 by KDIGO) (2). Lastly, in an exclusively neonatal cohort of 58 subjects who underwent cardiac surgery, with a median follow-up of 6 years, AKI was not associated with CKD or hypertension despite a high prevalence overall (17 and 30%, respectively) (29). Post-operative cyanosis was the only independent predictor of CKD.

Biomarkers have been explored as a way to detect kidney injury earlier than rising serum creatinine and to predict hospital outcomes in both adult and pediatric settings (33–37). These biomarkers are also being explored as possible markers of CKD and its progression (38). Two pediatric studies looked at the long-term changes in biomarkers, both in cardiac surgery populations. The Translational Research Investigating Biomarker End Points in AKI (TRIBE-AKI) consortium found that urinary neutrophil gelatinase-associated lipocalin (NGAL) and urinary interleukin-18 (IL-18) concentrations rose acutely postoperatively in children with AKI but then decreased by the 5-year follow-up (25). They did not find a difference in median urine biomarker levels in children with severe AKI at 5-year follow-up compared to non-AKI patients. Unlike the TRIBE-AKI study, the Follow-Up Renal Assessment of Injury Long-Term After Acute Kidney Injury (FRAIL-AKI) study demonstrated that 7–8 years after cardiac surgery, children with AKI had higher urinary IL-18, kidney injury molecule-1 (KIM-1), and liver-type fatty acid-binding protein (L-FABP) concentrations (24). Despite these changes in biomarker profiles, these studies have not reported higher risk associations for adverse kidney outcomes in children with AKI post-cardiac surgery.

### What Is New?

One of the major improvements in published studies since 2015 has been the standardization of AKI definitions, the inclusion of non-AKI comparison groups, and standardized definitions of hypertension, proteinuria, and CKD. The previous meta-analysis found an overall high prevalence of adverse kidney outcomes in AKI populations, however, more recent studies with non-AKI comparison groups and larger cohorts allow us to better understand this population's long-term risk (12). In the general PICU population, AKI is associated with an increased risk of CKD and hypertension compared to the non-AKI population after controlling for important confounders using both measured outcome data and administrative data. In the cardiac surgery population, intriguingly this risk association remains less clear, however as previously highlighted, this population has a high prevalence of kidney outcomes in long-term follow-up (12). Early research on the use of biomarkers for evaluating kidney recovery or CKD development has been published but validation of these biomarkers against gold standard measurements of kidney function are needed before they can be widely used.

## Healthcare Utilization

In adults, AKI is associated with an increased risk of rehospitalization. In this population, the KDIGO guidelines suggest follow-up care 3 months after an AKI episode to assess new onset or worsening CKD (i.e., assess serum creatinine, urine for protein, blood pressure) (39–41). Limited data have been published on the impact of AKI on long-term healthcare utilization in children following their initial admission to the PICU. Two studies reporting on healthcare utilization, one in a non-cardiac surgery population and the other in those following cardiac surgery, have been published (Table 3).

**Table 3:** Summary of studies reporting AKI and long-term healthcare utilization published over the past 5-years.

**References**	**Study design**	***N* patients**	**Inclusion criteria**	**AKI definition**	**Duration of follow up**	**Outcome definition**	**Findings**
Hessey et al. (5)	Two center retrospective cohort study	2,041	General PICU, non-cardiac surgery	KDIGO	5 years	30-day, 1-year, and 5-year hospitalizations, ED visits, and physician visits based on administrative data	• Patients with AKI (yes/no) and stage 2 or 3 AKI had increased risk of a 1- and 5-year hospitalization and 5-year physician visits • AKI was not associated with emergency department visit
Nunes et al. (18)	Multicenter prospective cohort study	402	Cardiac surgery	KDIGO	1-year	30-day and 1-year hospital readmission by chart review	• Patients with AKI did not have increased 30-day or 1-year readmission post-cardiac surgery compared to non-AKI patients • Stage 2 or 3 AKI was not associated with 30-day [aHR 1.5 (0.6–3.8)] or 1-year [aHR 0.98 (0.5–1.7)] readmission post-cardiac surgery

*Key articles evaluating the association between PICU-AKI and healthcare utilization published since 2015. This may not be an exhaustive list as a formal systematic review search was not performed*.

*KDIGO, Kidney Disease: Improving Global Outcomes; AKI, acute kidney injury; aHR, adjusted hazard ratio; PICU, pediatric intensive care unit; ED, emergency department*.

### General PICU Population

In the non-cardiac surgery PICU population, although there was no association with increased emergency department visits in those who developed AKI, children with AKI had a 35% higher risk of 1-year hospitalization, and 59% higher risk of 5-year hospitalizations compared to children without AKI (5). AKI was also associated with increased physician visits over 5 years [relative risk (95% CI) 1.12 (1.07–1.18)]. Importantly, <25% of the children with AKI saw a nephrologist in a 5-year follow-up (5). The reason for nephrology follow-up was not reported nor was a diagnosis of CKD or hypertension, however, this still highlights that even children with severe AKI in the PICU do not have regular follow-up with kidney specialists.

### Cardiac Surgery Population

In the cardiac surgery population, neither any AKI (i.e., stage 1, 2, or 3) nor severe AKI (stage 2 or 3 by KDIGO criteria) were associated with 30-day or 1-year hospital readmission (18). Unlike the aforementioned study in the non-cardiac population which evaluated the number of events per person-time (count data), this study focused on readmissions as a binary outcome. Therefore, further research evaluating cumulative use healthcare is required to better understand if there are differences in healthcare utilization in this population.

### What's New?

Evaluating the impact of AKI on long-term healthcare utilization is a new area of research that has emerged in the pediatric literature over the past 5 years. At this time, it remains unclear if AKI itself contributes to increased healthcare utilization in the general PICU population either by long-term kidney and non-kidney sequelae that may progress following AKI or if perhaps, AKI is a marker of a patient's medical complexity. Due to the limited data on healthcare utilization following AKI in the PICU, we are not able to draw strong conclusions at this time. Research comparing healthcare utilization prior to critical illness and after critical illness (i.e., a difference-in-difference approach), specifically evaluating AKI as a risk factor, would help to clarify this. Although there are currently no guidelines for post-discharge monitoring after pediatric AKI, non-cardiac surgery children who experience AKI whilst in PICU may require closer follow-up by primary care providers, hence leading to increased physician visits. In those following cardiac surgery, this is “in built” to their current care pathways with sub-specialist cardiac reviews and follow-up. Research on provider practice is needed to further evaluate this.

## Discussion

With the development of a standardized definition for AKI and increased awareness of long-term sequelae of AKI described in the adult literature, more research evaluating pediatric AKI and long-term outcomes has been reported (21, 39). In the general PICU population, AKI is associated with an increased risk of long-term mortality, healthcare utilization, CKD, and hypertension. In the cardiac surgery population, the association between AKI and long-term outcomes is less clear; however, these children remain at high risk of kidney sequelae compared to the general population. Since the meta-analysis by Greenberg et al. published in 2014, non-AKI comparison cohorts have been included in research studies allowing for the evaluation of AKI with long-term outcomes rather than simply making comparisons with the general pediatric population (12). This has not only allowed us to identify AKI as an important risk factor for poor long-term outcomes, but it has also allowed us to quantify the magnitude of risk of developing these outcomes.

Over the past 5 years, more data has been published beyond early mortality (within 28-days) describing both medium (1–2 year) (6, 15, 16, 18) and long-term mortality risk (>3 years) (17, 19). Importantly, only two studies evaluated post-discharge mortality (i.e., excluded hospital mortality from the index admission). While the focus of this review was on long-term outcomes, we included key studies evaluating 28-day mortality as we felt this data frames the population and demonstrates the differences in risk of mortality during the acute illness and the long-term. This data highlights potential future research directions and the need to focus on evaluating in-hospital vs. post-discharge mortality to improve our understanding of the timelines and etiologies of mortality risk in this heterogeneous population of children with hospital-acquired AKI.

There remains a divergence of reported long-term kidney outcomes in children who developed PICU-AKI. Larger cohorts with general PICU populations show a consistent association between PICU-AKI and hypertension, proteinuria, and CKD. In exclusively cardiac surgery populations this association is less clear and requires further investigation. It remains to be shown if this is a result of study cohort size and shorter follow-up duration, if it is due to differences in the patient population, or the management of the patients in the peri-operative period or subsequently in the immediate post-operative period. It is well-known that there is a high prevalence of AKI after cardiac surgery and this is associated with poor short-term outcomes. There may be more strategies in place to identify and mitigate post-operative declines in kidney function. This population of patients needs further evaluation in larger multi-center cohorts with longer follow-up duration to better determine the long-term risk of kidney sequelae.

Biomarkers are an important emerging area of research and may allow for earlier detection of both acute and chronic kidney problems. However, standardized methods for measuring biomarkers and validated CKD definitions showing biomarker level associations with gold standard GFR measurements in children are needed before they can be widely used. At this time, proteinuria is the urinary marker used to identify early kidney disease. Disappointingly, rates of proteinuria are not reported uniformly in studies evaluating long-term CKD. However, this is important information as monitoring albumin-to-creatinine ratio or protein-to-creatinine ratio in a child with previous AKI is a simple investigation, less invasive than serial serum creatinine measurements, and may detect early kidney disease in this population.

All the studies that evaluated the long-term risk of kidney outcomes report composite measures, which include some combination of hypertension, proteinuria, albuminuria, and reduced GFR. Although composite measures have been used in the literature, moving forward it is important to report kidney outcomes more systematically, categorizing by presence (yes/no) and where applicable, by level for proteinuria, albuminuria, hypertension, CKD, and reduced eGFR. Detailed reporting is likely to improve our understanding of kidney outcomes, as often CKD is used as a “blanket term.” Before these recent publications, there was little data on the prevalence of kidney outcomes in the pediatric AKI population, and these diagnoses are relatively uncommon in the general pediatric population. Therefore, early studies likely used composite measures to first assess if there was an association between AKI and long-term kidney outcomes. With the data showing increased prevalence of kidney outcomes in this population, future studies should aim to report these data with improved granularity using consensus definitions.

With many medical systems transitioning to electronic medical records, we are beginning to see larger population-based studies emerge in the pediatric AKI literature (6). While prospective follow-up studies provide exceptional information, these are labor-intensive and costly, and the retention of patients can be a challenge. Using administrative data is a cost-effective method for examining long-term outcomes in a large population. A systematic review in adults has demonstrated that AKI is identified by administrative health data with low sensitivity, but high specificity; similar results were also obtained from a validation study for identifying AKI in the PICU (42, 43). Adult studies have used databases to evaluate the association between AKI and long-term CKD development (22). In the pediatric literature, studies have started exploring the use of administrative data; however, the algorithms used to define CKD and hypertension have not yet been validated (26, 28). A validation study of long-term kidney outcome definitions with administrative data would allow for much larger population based studies with longer follow-up times. Validation studies in adults have demonstrated low sensitivity of these diagnostic code algorithms which is a major limitation (44, 45). These administrative data definitions seem to identify the more severe disease and therefore are likely to underestimate the prevalence of outcomes. It is important to identify the population of patients with early CKD or mild proteinuria, as early intervention can slow the progression of the disease. In the future, as more electronic medical record databases become available, combining what we have learned from administrative database studies with patient measurements will allow for larger population studies with more sensitive outcome measurements.

Recovery from AKI has also been an important variable to monitor in this population and has been associated with long-term CKD development in children (46). Unfortunately, in retrospective studies, this can be difficult to assess as many patients with AKI do not have repeat creatinine measurements (47). At this time there is no standardized definition for kidney function recovery and therefore studies evaluating AKI recovery with outcomes can be difficult to compare and interpret. Further research is needed to examine the association between incomplete recovery after AKI and long-term outcomes. Various definitions should be evaluated in these studies to better determine what level of incomplete recovery puts children at higher risk. This information would be invaluable for long-term follow-up guideline development and risk stratification.

As our detection and treatment of various illnesses in the PICU improves, so does PICU survival; however, this may also be associated with greater long-term morbidity (48). At this time, it remains unclear whether the association between AKI and long-term mortality and healthcare utilization is related to the direct effect of kidney damage or if AKI is a marker of medical complexity. This also means further exploring the impact AKI has on long-term health-related quality of life (HRQoL). One study in children with sepsis in the PICU showed that children with severe AKI (stage 2 or 3 by KDIGO) had poorer HRQoL at 3-months than children with no AKI or stage 1 AKI (49). Future research is needed to further assess the relationship between AKI and long-term markers of morbidity. These studies should also evaluate other clinical factors that may contribute, to allow for more targeted risk stratification models and follow-up guidelines.

There are many important outcomes that have been explored in the adult AKI literature that remain as gaps in knowledge in the pediatric population. Specifically, there are validated administrative data definitions for kidney outcomes, more evidence on recovery after AKI as well as recurrent AKI events, and measures of quality of life (44, 45, 50, 51). Adult studies have also shown that AKI increases direct and indirect healthcare costs and resource utilization (50). These outcomes remain as gaps in our current pediatric AKI research and warrant further attention. The use of standardized AKI definitions and non-AKI comparison groups should also be continued in all future pediatric AKI research.

Overall, it is clear from the literature that children with PICU associated AKI have an increased risk of adverse medium and long-term outcomes including mortality. Future challenges include standardizing our approach to the recognition and management of these patients beyond the critical care and nephrology healthcare professionals. It is important therefore to have the diagnosis of AKI documented when the patient is discharged from the PICU and subsequently from the hospital so that both non-critical care professionals who are hospital-based and those outside the hospital know to monitor these children more closely for resolution of AKI and for long-term sequelae. This would also allow providers to make more educated decisions about future treatments, including avoiding nephrotoxic medications where possible and providing lifestyle modification advice. To date, outlines of possible follow-up guidelines have been suggested but no formal guidelines exist (52, 53). Adult AKI guidelines suggest assessing the resolution of AKI 3 months after the initial insult (39). As highlighted in this review, children who develop severe AKI in the PICU are at increased risk of adverse kidney and non-kidney outcomes, so similar follow-up recommendations are required.

The 22nd Acute Disease Quality Initiative (ADQI) conference recently published quality improvement recommendations for pediatric AKI (46). This consensus statement recommends kidney health assessment every 1–2 years, which includes an AKI history, blood pressure measurement, serum creatinine measurement, and drug list review, in high-risk populations which include patients admitted to the PICU. They also recommended kidney health assessment following an unplanned acute exposure such as PICU admission (46). These recommendations need evaluation for implementation in different health settings but are a welcome expert consensus statement that is likely to mitigate adverse outcomes in this high-risk population. One of the challenges of developing AKI guidelines in this population remains that all children with AKI cannot be followed by nephrologists alone both because of large numbers of patients (6) but also because of the increasing burden of healthcare visits on children and families. Therefore, a coordinated effort must be made between general pediatricians and practitioners that follow these children in the community and the discharging hospitalist or specialists. While future research will continue to help us better understand additional risk factors for long-term outcomes and develop better risk stratification models, it is clear that children with PICU-AKI require closer follow-up. Early CKD and hypertension are treatable and timely detection and intervention will improve outcomes in these children.

## Author Contributions

EH conceptualized the review, performed the literature review, and drafted and edited the manuscript. NM conceptualized the review, assisted with the literature review, drafted and edited the manuscript, and provided expert knowledge. MS conceptualized the review, assisted with the literature review, edited the manuscript, and provided expert knowledge. RA assisted with the literature review, edited the manuscript, and provided expert knowledge. EU, CM, and SB provided expert knowledge and edited the manuscript. All authors agreed to the final submitted manuscript.

## Conflict of Interest

The authors declare that the research was conducted in the absence of any commercial or financial relationships that could be construed as a potential conflict of interest.
